# Effect of Fluorescence Visualization–Guided Surgery on Local Recurrence of Oral Squamous Cell Carcinoma: A Randomized Clinical Trial

**DOI:** 10.1001/jamaoto.2020.3147

**Published:** 2020-10-08

**Authors:** J. Scott Durham, Penelope Brasher, Donald W. Anderson, John Yoo, Rob Hart, Joseph C. Dort, Hadi Seikaly, Paul Kerr, Miriam P. Rosin, Catherine F. Poh

**Affiliations:** 1 Department of Surgery, Faculty of Medicine, The University of British Columbia, Vancouver, British Columbia, Canada; 2 Department of Statistics, Faculty of Science, The University of British Columbia, Vancouver, British Columbia, Canada; 3 Department of Otolaryngology–Head and Neck Surgery and Oncology, London Health Sciences Center, London, Ontario, Canada; 4 Division of Otolaryngology–Head and Neck Surgery, University of Calgary, Calgary, Alberta, Canada; 5 Division of Otolaryngology, Department of Surgery, Faculty of Medicine and Dentistry, University of Alberta, Edmonton, Alberta, Canada; 6 Department of Otolaryngology–Head & Neck Surgery, Rady Faculty of Health Sciences, University of Manitoba, Winnipeg, Manitoba, Canada; 7 School of Biomedical Physiology and Kinesiology, Simon Fraser University, Burnaby, British Columbia, Canada; 8 Department of Oral Biological and Medical Sciences, Faculty of Dentistry, The University of British Columbia, Vancouver, British Columbia, Canada

## Abstract

**Importance:**

High local recurrence rates with aggressive disease remain the main concern in oral cancer survival. Use of a translational device using fluorescence visualization (FV) approved by the US Food and Drug Administration and Health Canada, has shown a marked reduction in the 3-year local recurrence rate of high-grade oral lesions in a single-center observational study.

**Objective:**

To determine whether FV- guided surgery can improve local control rates in the treatment of in situ or T1 to T2 category oral squamous cell carcinoma (OSCC).

**Design, Setting, and Participants:**

A multicenter randomized clinical trial was conducted in a surgical setting. A total of 457 patients were enrolled between January 18, 2010, and April 30, 2015. Data analysis of the intention-to-treat population was performed from April 3, 2019, to March 20, 2020. Patients with histologically confirmed high-grade dysplasia/carcinoma in situ or T1 to T2 category OSCC were randomized to receive traditional peroral surgery or FV-guided surgery.

**Intervention:**

Fluorescence visualization during surgery.

**Main Outcomes and Measures:**

The primary outcome was local recurrence of OSCC. Secondary outcomes were failure of the first-pass margin, defined as a histologically confirmed positive margin for severe dysplasia or greater histologic change of the main specimen (ie, not the margins taken from the resection bed), regional or distant metastasis, and death due to disease.

**Results:**

Of the 457 patients enrolled in the study, 443 patients (264 [59.6%] men; mean [SD] age, 61.5 [13.3] years) completed the randomized treatment: 227 FV-guided and 216 non-FV guided surgery. The median follow-up was 52 (range, 0.29-90.8) months. In total, 45 patients (10.2%) experienced local recurrence. The 3-year local recurrence rate was 9.4% in the FV-guided group and 7.2% in the non-FV group (difference, 2.2%; 95% CI, −3.2% to 7.4%). Other similarities between the FV vs non-FV groups included failure of first-pass margin (68/227 [30.0%]) vs 65/216 [30.1%]), regional failure (39/227 [17.2%] vs 37/216 [17.1%]), disease-specific survival (23/227 [10.1%] vs 19/26 [8.8%]), and overall survival (41/227 [18.1%] vs 38/216 [17.6%]) were also similar between groups. No adverse events were judged to be related to the intervention.

**Conclusions and Relevance:**

In this randomized clinical trial, FV-guided surgery did not improve local control rates in the treatment of patients with in situ or T1 to T2 category oral cancer. Under a controlled environment, FV-guided surgery did not have an evident effect in reduction of local recurrence for localized OSCC. This result suggests that attention be directed to strategies other than improving definitions of nonapparent disease at clinical margins to identify the sources of local recurrence.

**Trial Registration:**

ClinicalTrial.gov Identifier: NCT01039298

## Key Points

**Question:**

Is the use of fluorescence visualization (FV) during surgery associated with reduced local recurrence of localized oral squamous cell carcinoma?

**Findings:**

In this multicenter randomized clinical trial of 443 patients, compared with those who underwent non–FV-guided conventional surgery, 227 patients who received FV-guided surgery had no significant difference in 3-year local recurrence.

**Meaning:**

In this study, under a controlled environment, patients with localized oral squamous cell carcinoma who underwent FV-guided surgery had a similar risk of local recurrence to those who received conventional non–FV-guided surgery.

## Teaser

This randomized clinical trial compares the effect of fluorescence visualization– vs white light–guided surgery on local recurrence rates in patients with oral squamous cell carcinoma.

## Introduction

Oral cancer remains a significant disease burden with minimal improvement in survival over 5 decades.^1^ High local recurrence rates with the aggressive disease remain the main concern.^2,3^ It has been reported that genetically altered cells are often widespread across the mucosa of patients with oral cancer, beyond clinically and histologically healthy tissue,^4,5^ and that occult disease frequently extends beyond the tumor clearance area and, consequently, may be responsible for the high rate of cancer recurrence at the primary site (10%-30% of patients).^6,7,8^ Development of new approaches that can be adopted easily in clinical settings to facilitate the detection of clinically occult fields with a high risk for oral cancer remains a need.

A fluorescence visualization (FV) tool has been developed to improve the direct visualization of a clinically normal-looking field surrounding a visible oral cancer.^9,10^ The altered fields beyond the clinically apparent tumor have been shown to carry either high-grade pathologic characteristics or high-risk molecular clones that might be associated with high local recurrence.^9^ In a single-center observational study, a marked reduction was noted in the 3-year local recurrence rate of high-grade lesions (HGLs) (ie, severe dysplasia or carcinoma in situ) and oral squamous cell carcinoma (OSCC) with FV-guided surgical excision.^11^ We conducted a multicenter randomized clinical trial to compare white light (WL)- and FV-guided surgery in the treatment of HGLs and localized T-category OSCC FV-guided surgery.^12^

## Methods

This parallel group, multicenter randomized clinical trial compared WL- and FV-guided surgery. Parts of the study protocol were published previously^12^ and the full version is available in Supplement 1. Approval for the study was obtained from the research ethics board at each of 7 Canadian centers (University of British Columbia Cancer Agency Research Ethics Board, Vancouver, British Columbia; Conjoint Health Research Ethics Board, Calgary, Alberta; Alberta Health Services, Edmonton, Alberta; University of Manitoba Health Research Ethics Board, Winnipeg, Manitoba; University of Western Ontario Health Services Research Ethics Board, London, Ontario; Sunnybrook Health Sciences Center Research Ethics Board, Toronto, Ontario; and Capital Health Research Ethics Board, Halifax, Nova Scotia). All patients provided written informed consent. Overall study coordination and data processing were done at the central site (BC Cancer, Vancouver, British Columbia). This study followed the Consolidated Standards of Reporting Trials (CONSORT) reporting guideline for randomized clinical trials.

### Study Population

Patients were eligible for the study if they had a histologically confirmed diagnosis of preinvasive HGL or localized T-category (T1 or T2) invasive OSCC, based on the *American Joint Committee on Cancer, 8th Edition* (*AJCC 8*), guidelines,^13^ either de novo or with a history of HGL or OSCC. If the lesion was a local recurrence, the patient had to be at least 6 months posttreatment so as not to impact lesion visualization. Patients with the following conditions were excluded: concurrent nonoral cancer diagnosed within 3 years (nonmelanoma skin cancer or lymphoma outside of the head and neck region were included in the study), evidence of distant metastasis, any illness that could preclude standard diagnostic tests and postsurgery follow-up, or lesions at the base of the tongue or tonsil. The demographic and risk factor data were given by the patients and collected by participating clinicians using a standard questionnaire across all study centers (Supplement 1). Data on ethnicity were collected to inform the proportion of ethnic groups in the study population.

Participating surgeons identified potentially eligible patients at the time of their surgical consultation and briefly introduced the study. Patients who expressed an interest in study participation attended the clinic for a presurgical assessment by the local FV specialist (FVS), who was a different surgeon or dentist from the participating surgeon, and the research coordinator (RC), which included a computed tomographic scan of the head, neck, and chest to rule out metastases and confirm clinical nodal status. Once eligibility was confirmed and consent obtained, the patient completed the baseline questionnaires. The local RC worked with the surgeon’s office to identify the date of surgery. A few days before the scheduled surgery, the central RC would access the web-based, study-specific randomization program to determine treatment allocation. Once the allocation was determined, the center RC would notify the local RC and the local FVS of the assignment. Randomization was stratified by center and histologic grade of the primary lesion (severe dysplasia/carcinoma in situ, invasive SCC). Furthermore, within each stratum, a minimization algorithm was used to achieve balance with respect to the surgeon, sex, age, smoking history, and anatomic site.

### Treatment and Follow-up

Patients were prepared for surgery following the local standards of care. For both groups, at the time of surgery, the operating surgeon outlined the boundary of the clinically visible lesion under WL. The surgeon then left the room while the FVS, with the operating room lights off, used a green Sharpie pen to either draw an outline on top of the surgeon's clinical boundary (WL-guided group) or used the FV instrument (VELscope Vx, Apteryx Imaging) to examine the lesion and draw an outline around the area of FV change (FV-guided group). The surgeon then returned to the operating room and outlined a 10-mm surgical boundary around both the clinical and FV boundaries, whichever was wider; surgical excision of the marked area followed. The local study pathologist was notified, and the surgical specimens were pinned, imaged, and processed as described previously.^11^

Clinic visits occurred every 3 months for 2 years following surgery and then every 6 months in years 3 to 5. At each visit, the oral mucosa was examined under WL, and photographs of the surgical sites were taken. For possible recurrent disease, the decision for biopsy or computed tomographic scanning was left to the discretion of the surgeon. If the patient remained free of recurrence, a comparative biopsy at the surgical site was taken 24 months postsurgery.

### Mitigation of Bias

Central randomization and the incorporation of a minimization method ensured that treatment allocation was concealed from all parties. Only the local RC and the central data manager had access to the study randomization list. The FVS was aware of the treatment assignment but played no part in the treatment or follow-up of the patients. It is unavoidable that the surgeon could determine the treatment assignment by comparing the 2 marked boundaries. To mitigate deviation from the marked boundary, photographs of the boundaries were taken in the operating room just before surgery and, once every 3 months at the first year of the trial, a random sample of cases (5%) from each study site was reviewed to check for the accuracy of boundary drawings.

### Outcomes

The primary outcome was local recurrence, defined as a recurrence at or within 1 cm of the surgical site, with the same or a higher grade of histologic abnormality than the initial diagnosis or further treatment owing to the presence of severe dysplasia or higher degree of change at follow-up. Secondary outcomes were (1) failure of the first-pass margin, defined as a histologically confirmed positive margin for severe dysplasia or greater histologic change of the main specimen, not the margins taken from the resection bed; (2) regional metastasis, defined as nodal positive either identified from elective neck dissection at the time of surgery or from postsurgery follow-up, or distant metastasis; (3) death due to disease; and (4) quality of life as measured by the Health-Related Quality of Life EQ-5D,^14,15^ the Functional Assessment of Cancer Therapy–Head & Neck^16,17^ and the Speech Handicap Index.^18^

A data and safety monitoring board met every 6 months to monitor accrual and review adverse events. Two interim analyses for efficacy were planned, after one-third and two-thirds of predicted local recurrences. A Lan-DeMets spending function with O'Brien-Fleming stopping boundaries was to be used to control for multiple testing.^19^ The final analysis was to be conducted 2 years after the last patient was enrolled.

### Statistical Analysis

The target sample size was 400 (240 invasive SCC and 160 severe dysplasia/cancer in situ). Assuming exponential survival distributions, an overall 25% failure rate at 3 years in the WL-guided arm, a 36-month accrual period, a minimum of 2 years of follow-up, and a type I error of .045 for the final analysis, we required a total sample size of 350 patients to detect a 50% reduction in local recurrence at 3 years with at least 80% power. The anticipated number of events was 104. We increased the sample size to 400 to allow for dropouts and deaths prior to local recurrence. Intention-to-treat analysis was conducted.

Baseline data were described by treatment group; continuous data were summarized as mean (SD) or median (first, third quartiles) if the data were skewed; frequencies and proportions summarized categorical data. Times to event were measured from the date of surgery. In the analyses we used the cumulative incidence estimator and proportional subdistribution hazard model described by Fine and Gray^20^ to account for competing risks. Data analysis of the intention-to-treat population was performed from April 3, 2019, to March 20, 2020. All analyses (unpaired, 2-sided; *P* < .05 considered significant) were performed using Stata, version 14.2,^21^ and R, version 3.5.0.^22^

## Results

Enrollment began at the central coordinating center on January 18, 2010, with expansion to the other centers beginning on September 1, 2010. An interim analysis was performed on November 20, 2014, after the target sample size had been reached. The interim analysis found a combined cumulative incidence for local recurrence of 10% at 36 months, which was substantially lower than had been anticipated. The data and safety monitoring board recommended continuing recruitment and extending follow-up to 5 years. Enrollment ended on April 30, 2015, when the funding ended, with a total of 457 participants. The final clinic visit occurred on October 31, 2017.

**Figure 1.**
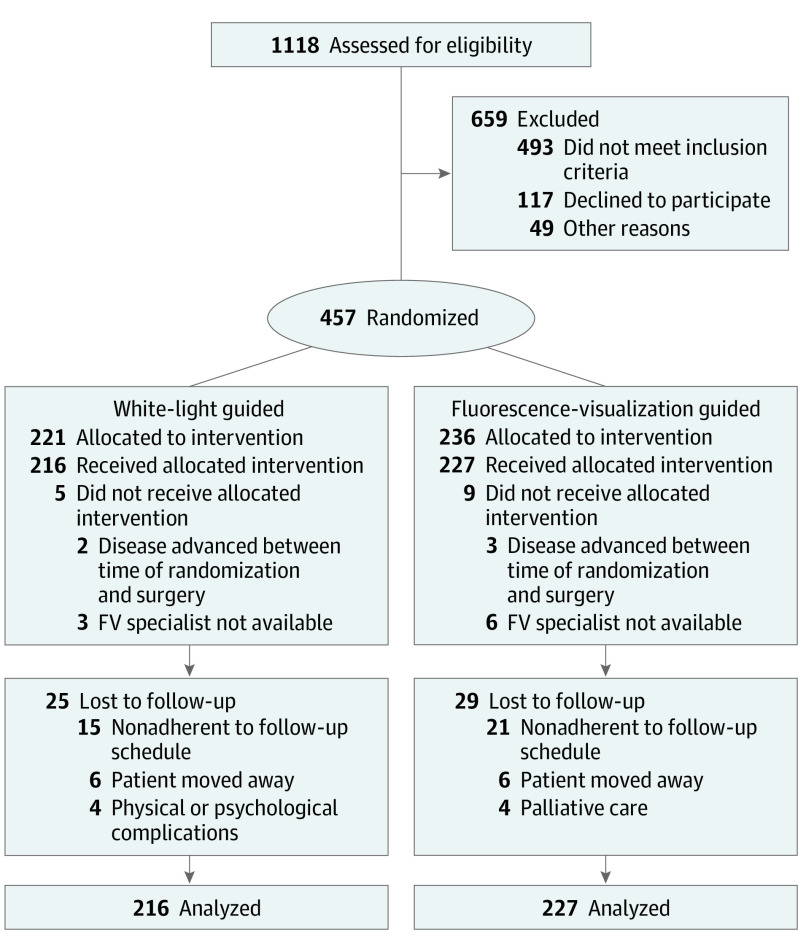
CONSORT Flow Diagram FV indicates fluorescence visualization.

The flow of study participants is shown in Figure 1. Fourteen patients did not receive their randomized treatment owing to logistical issues (n = 9) or because they were found to be ineligible owing to disease advancement at the time of surgery (n = 5). Table 1 describes the remaining 443 study participants; a total of 264 men (59.6%) and 179 women (40.4%) were included and mean (SD) age was 61.5 (13.3) years. The 2 groups had similar baseline characteristics. Of the 261 OSCC cases, 52 patients (19.9%) received postsurgery radiotherapy or chemoradiotherapy; of these, none had a local recurrence. In addition, more patients in the FV-guided arm (10/227 [4.4%]) vs the WL-guided arm (3/216 [1.4%]) received adjuvant radiotherapy or chemoradiotherapy. Follow-up (median, 52 [range, 0.29-90.8] months) was similar in the 2 groups overall and with regard to the primary time point of interest (3 years); approximately 9% (WL, 17 and FV, 21 participants) were lost to follow-up at the 3-year point (3% had 1 year of follow-up and 5% had 2 years of follow-up), and an additional 56 patients (12.6%) died before the 3-year mark without experiencing the primary end point (Table 2).

**Table 1.** Baseline Characteristics

Characteristic	No. (%)		
	Total (n = 443)	WL guided (n = 216)	FV guided (n = 227)
Age, mean (SD), y	61.5 (13.3)	61.4 (12.9)	61.5 (13.6)
Sex			
Men	264 (59.6)	128 (59.3)	136 (59.9)
Women	179 (40.4)	88 (40.7)	91 (40.1)
Ethnicity			
Aboriginal	4 (0.9)	3 (1.4)	1 (0.4)
Asian	65 (14.7)	31 (14.3)	34 (15.0)
White	371 (83.7)	181 (83.8)	190 (83.7)
Other	3 (0.7)	1 (0.5)	2 (0.9)
Type			
HGL	182 (41.1)	89 (41.2)	93 (41.0)
SCC^a^	261 (58.9)	127 (58.8)	134 (59.0)
T1	115 (26.0)	53 (24.5)	62 (27.3)
T2	146 (32.9)	74 (34.2)	72 (31.7)
Smoking status			
Never	154 (34.8)	80 (37.0)	74 (32.6)
Former	148 (33.4)	66 (30.6)	82 (36.1)
Current	141 (31.8)	70 (32.4)	71 (31.3)
Lymph node involvement	20 (4.5)	12 (5.6)	8 (3.5)
Oral cancer history			
None	389 (87.8)	190 (88.0)	199 (87.7)
Previous HGL	20 (4.5)	11 (5.1)	9 (4.0)
Previous SCC	34 (7.7)	15 (6.9)	19 (8.4)

Abbreviations: FV, fluorescence visualization; HGL, high-grade lesion; SCC, squamous cell carcinoma; WL, white light.

^a^ Based on *American Joint Committee on Cancer, 8th Edition*.^13^ T1, primary tumor size of 2 cm or less in greatest dimension; T2, primary tumor size more than 2 cm but not more than 4 cm in greatest dimension.

**Table 2.** Events and Adequacy of Follow-up for Primary Outcome of Local Recurrence

Outcome	No. (%)		
	Total (n = 443)	WL guided (n = 216)	FV guided (n = 227)
Primary outcome			
Local recurrence	45 (10.2)	21 (9.7)	24 (10.6)
Secondary outcomes			
Failure of first-pass margin	133 (30.0)	65 (30.1)	68 (30.0)
Regional failure			
At time of surgery	34 (7.7)	18 (8.3)	16 (7.0)
Postsurgery	42 (9.5)	19 (8.8)	23 (10.3)
Died, all causes^a^	79 (17.8)	38 (18.0)	41 (18.1)
Died of disease	42 (9.5)	19 (8.8)	23 (10.1)
Died of other causes	37 (8.4)	19 (8.8)	18 (7.9)
Adequacy of follow-up for local recurrence			
Died without local recurrence at <36 mo	56 (12.6)	29 (13.4)	27 (11.9)
≥36 mo of follow-up^b^	306 (69.1)	154 (71.3)	152 (66.9)
Follow-up time, median (IQR), mo^c^	54.0 (34.9-60.4)	55.2 (36.6-61.1)	51.7 (34.0-51.7)

Abbreviations: FV, fluorescence visualization; IQR, interquartile range; WL, white light.

^a^ Died of disease includes 14 deaths due to distant metastasis (WL, 8; FV, 6); died of other causes includes 24 deaths before 36 months (WL, 11; FV, 13).

^b^ Excludes those who experienced a local recurrence before 36 months.

^c^ Excludes those with a local recurrence at any time.

**Figure 2.**
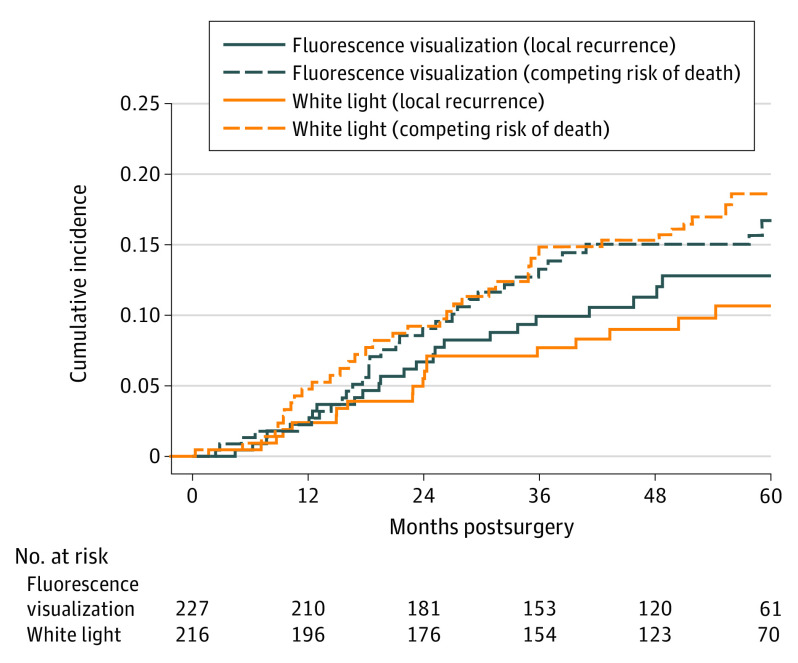
Cumulative Incidence Curves for Local Recurrence The dashed lines represent competing risk of death for fluorescence visualization–guided and white light–guided treatment groups.

**Figure 3.**
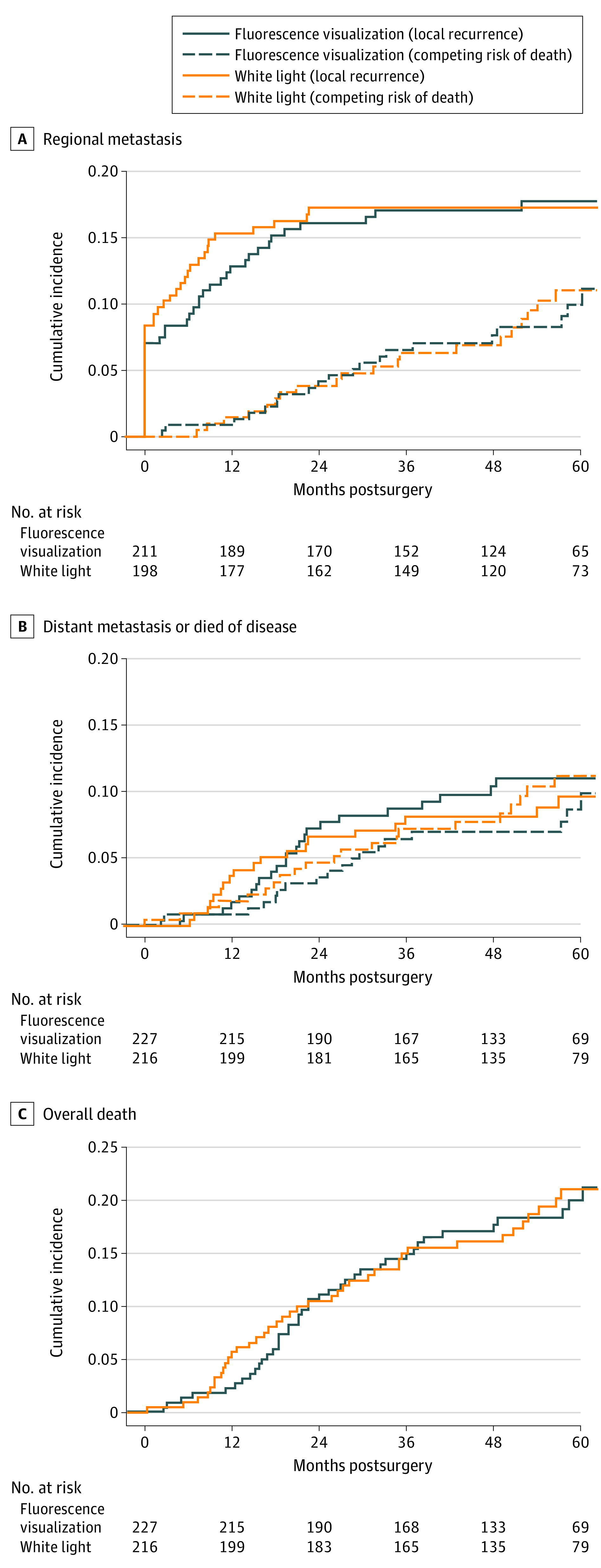
Cumulative Incidence Curves for Secondary Outcomes A, Regional; B, Distant failure or died of disease; C, All deaths. Dashed lines represent competing risk of death and death from other causes in the fluorescence visualization–guided and white light–guided treatment groups.

Forty-five patients (10.2%) experienced a local recurrence during follow-up (SCC, 25/261 [9.6%]; HGL, 20/182 [11.0%]). There was no statistically significant difference between the FV- vs WL-guided groups (HR, 1.13; 95% CI, 0.63-2.03). The estimated cumulative incidence for local recurrence at 3 years postsurgery was 9.4% in the FV-guided group and 7.2% in the WL-guided group (Figure 2), with a difference of 2.2% (95% CI, −3.2% to 7.4%). There was no significant difference in failure of first-pass margin between FV-guided (68/227 [30.0%]) and WL-guided (65/216 [30.1%]) groups. There were 34.6% (46 of the 133 failed first-pass margin) positive for deep margin and there was no significant difference between the 2 groups (11 [16.9%] vs 13 [19.1%]). According to the definition in the *AJCC 8*, among 146 of 261 cases (55.9%) of T2 category SCC, there were 13 T2 category (8.9%) cases with a positive deep margin, compared with only 5 cases (4.3%) with a positive deep margin among T1 category cases. Although not statistically significant, there were more T2 cases with failed deep margin. Regional failure (39/227 [17.2%] vs 37/216 [17.1%]), disease-specific survival (23/227 [10.1%] vs 19/26 [8.8%]), and overall survival (41/227 [18.1%] vs 38/216 [17.6%]) were also similar between groups (Figure 3).

### Adverse Events

Overall, 192 patients (89.9%) in the WL-guided arm and 200 patients (88.1%) in the FV-guided arm reported 1 or more adverse events, with oral pain (grade 1 or 2) being the most common (160/216 [74.1%] in WL-guided; 163/227 [71.8%] in FV-guided cohort) (eTable in Supplement 2).

Serious adverse events (grades 3, 4, or 5) were reported by 19.0% of patients (n = 41) in the WL-guided arm and 23.8% of patients (n = 54) in the FV-guided arm, with visits to the emergency department being most commonly reported (7.4% in both groups). There was no statistically significant difference between the treatment groups in the types of serious adverse events. No adverse events were judged to be specifically related to the intervention of FV-guided surgery.

## Discussion

To our knowledge, this is the first multicenter randomized trial to assess the efficacy of a translational tool (VELscope Vx) approved by the US Food and Drug Administration and Health Canada, in the control of local recurrence postsurgery. Among the 443 study participants, 91% completed 3-year follow-up with similar distribution between the 2 groups. This study demonstrated that FV-guided surgery was no more effective than WL-guided surgery in reducing local recurrence in patients with localized in situ or early T-category oral cancer. There were also no significant differences in the failure of first-pass margin, regional failure, disease-specific survival, or overall mortality.

The findings of low overall local recurrence rate and no significant difference in local recurrence between the groups were unexpected given the results of a previous observational study.^11^ Possible explanations for the overall low local recurrence rates are that the excision margins for the HGLs may have been more aggressive using the standardized protocol of 10-mm clearance, and more frequent postsurgery adjuvant intervention was used, with one-fifth of patients with OSCC receiving radiotherapy or chemoradiotherapy; of these, none had a local recurrence. In addition, higher-than-expected death rates (13% patients died without experiencing the primary end point) may also contribute to the low overall local recurrence rate. With respect to the small difference in local recurrence between the 2 groups, we considered several possibilities. First, the relative inexperience with FV of the sites outside the coordinating center potentially led to no effect of FV. Second, more patients in the FV-guided arm vs the WL-guided arm received adjuvant radiotherapy or chemoradiotherapy (none of these cases recurred during follow-up). In addition, more aggressive resection in the WL-guided group compared with the historical study might also explain the lack of difference.

### Limitations

The study has limitations. First, 14 patients did not receive their allocated treatment and are not included in the study. Five patients were found to be ineligible at the time of surgery; for the other 9 patients, the rescheduling of surgery without notification of the local RC meant the FVS was not available at the time of surgery. However, it is unlikely the exclusions have biased our results.^23^ Second, surgeons were likely aware of the treatment assignment at the time of lesion excision; however, in the operating room, we had additional observers (RC and FVS) to determine the final surgical margins and step-by-step photo documentation for the margins to avoid margin creep. Third, the lower-than-expected event rate, combined with the higher-than-expected death rate, resulted in fewer events than anticipated, leading to wide CIs for the estimates of the difference between treatments. However, the limits of the CI for the cumulative incidence of local recurrence at 3 years rule out any substantial benefit of FV- over WL-guided surgery.

In addition, FV is designed to evaluate the mucosal extent of tumor and adjacent at-risk mucosa. The deep tumor margin is often the most challenging to evaluate and clear at the time of surgery. We did not see the difference of observed deep margin between the 2 groups. Further research to prove real-time assessment on the adequacy of the deep margin is needed to resolve this clinically relevant issue.

One finding from this study relates to the surgical margins for HGL. High rates of persistence, recurrence, and eventual progression of such lesions to invasive SCC have led to general agreement that the lesions need to be removed. However, no agreement exists on the width of surgical clearance.^24,25^ We used 10-mm margins for all lesions and showed similar local recurrence rates for HGL and SCC. This finding provides evidence that some HGLs clinically behave like SCC and should be treated as aggressively as SCC.

## Conclusions

The results of this randomized clinical trial show that FV-guided surgery was no more effective than WL-guided surgery in the treatment of patients with localized in situ or T-stage oral cancer. To improve local recurrence, attention should be directed to strategies other than improving definitions of nonapparent disease at clinical margins.

## Notes

Supplement 1. Trial Protocol

Supplement 2. eTable. Adverse Events, Number of Patients (Events)

Supplement 3. Data Sharing Statement

## References

[1] Ferlay J, Soerjomataram I, Dikshit R, Cancer incidence and mortality worldwide: sources, methods and major patterns in GLOBOCAN 2012. Int J Cancer. 2015;136(5):E359-E386. doi:10.1002/ijc.29210 25220842

[2] Mariotto AB, Rowland JH, Ries LA, Scoppa S, Feuer EJ Multiple cancer prevalence: a growing challenge in long-term survivorship. Cancer Epidemiol Biomarkers Prev. 2007;16(3):566-571. doi:10.1158/1055-9965.EPI-06-0782 17372253

[3] Dhooge IJ, De Vos M, Van Cauwenberge PB Multiple primary malignant tumors in patients with head and neck cancer: results of a prospective study and future perspectives. Laryngoscope. 1998;108(2):250-256. doi:10.1097/00005537-199802000-00017 9473077

[4] Braakhuis BJ, Tabor MP, Kummer JA, Leemans CR, Brakenhoff RH A genetic explanation of Slaughter’s concept of field cancerization: evidence and clinical implications. Cancer Res. 2003;63(8):1727-1730. 12702551

[5] Slaughter DP, Southwick HW, Smejkal W Field cancerization in oral stratified squamous epithelium; clinical implications of multicentric origin. Cancer. 1953;6(5):963-968. doi:10.1002/1097-0142(195309)6:5<963::AID-CNCR2820060515>3.0.CO;2-Q 13094644

[6] Leemans CR, Tiwari R, Nauta JJ, van der Waal I, Snow GB Recurrence at the primary site in head and neck cancer and the significance of neck lymph node metastases as a prognostic factor. Cancer. 1994;73(1):187-190. doi:10.1002/1097-0142(19940101)73:1<187::AID-CNCR2820730132>3.0.CO;2-J 8275423

[7] Brennan JA, Mao L, Hruban RH, Molecular assessment of histopathological staging in squamous-cell carcinoma of the head and neck. N Engl J Med. 1995;332(7):429-435. doi:10.1056/NEJM199502163320704 7619114

[8] Tabor MP, Brakenhoff RH, van Houten VM, Persistence of genetically altered fields in head and neck cancer patients: biological and clinical implications. Clin Cancer Res. 2001;7(6):1523-1532. 11410486

[9] Lane PM, Gilhuly T, Whitehead P, Simple device for the direct visualization of oral-cavity tissue fluorescence. J Biomed Opt. 2006;11(2):024006. doi:10.1117/1.2193157 16674196

[10] Guillaud M, Zhang L, Poh C, Rosin MP, MacAulay C Potential use of quantitative tissue phenotype to predict malignant risk for oral premalignant lesions. Cancer Res. 2008;68(9):3099-3107. doi:10.1158/0008-5472.CAN-07-2113 18451134 PMC2693059

[11] Poh CF, Anderson DW, Durham JS, Fluorescence visualization-guided surgery for early-stage oral cancer. JAMA Otolaryngol Head Neck Surg. 2016;142(3):209-216. doi:10.1001/jamaoto.2015.3211 26769431

[12] Poh CF, Durham JS, Brasher PM, Canadian Optically-Guided Approach for Oral Lesions Surgical (COOLS) trial: study protocol for a randomized controlled trial. BMC Cancer. 2011;11:462. doi:10.1186/1471-2407-11-462 22026481 PMC3226575

[13] Amin MB, Edge S, Greene F, AJCC Cancer Staging Manual. 8th ed. Springer International Publishing: American Joint Commission on Cancer; 2017.

[14] Euro-Qol. EQ-5D Instruments. 2019 Accessed September 30, 2019. https://euroqol.org/euroqol/

[15] Shaw JW, Johnson JA, Coons SJ US valuation of the EQ-5D health states: development and testing of the D1 valuation model. Med Care. 2005;43(3):203-220. doi:10.1097/00005650-200503000-00003 15725977

[16] FACIT.org. FACIT classification overview 2019 Accessed September 30, 2019. https://www.facit.org/FACITOrg

[17] List MA, D’Antonio LL, Cella DF, The performance status scale for head and neck cancer patients and the functional assessment of cancer therapy—head and neck scale. a study of utility and validity. Cancer. 1996;77(11):2294-2301. doi:10.1002/(SICI)1097-0142(19960601)77:11<2294::AID-CNCR17>3.0.CO;2-S 8635098

[18] Rinkel RN, Verdonck-de Leeuw IM, van Reij EJ, Aaronson NK, Leemans CR Speech Handicap Index in patients with oral and pharyngeal cancer: better understanding of patients’ complaints. Head Neck. 2008;30(7):868-874. doi:10.1002/hed.20795 18302270

[19] DeMets DL, Lan KK Interim analysis: the alpha spending function approach. Stat Med. 1994;13(13-14):1341-1352. doi:10.1002/sim.4780131308 7973215

[20] Fine JP, Gray RJ A proportional hazards model for the subdistribution of a competing risk. J Am Statistical Assoc. 1999;94(446):496-509. doi:10.1080/01621459.1999.10474144

[21] Stata Statistical Software: release 14. StataCorp LP; 2015.

[22] Team RC. R: A Language and Environment for Statistical Computing. R Foundation for Statistical Computing; 2018.

[23] Fergusson D, Aaron SD, Guyatt G, Hébert P Post-randomisation exclusions: the intention to treat principle and excluding patients from analysis. BMJ. 2002;325(7365):652-654. doi:10.1136/bmj.325.7365.652 12242181 PMC1124168

[24] Schoelch ML, Sekandari N, Regezi JA, Silverman S Jr Laser management of oral leukoplakias: a follow-up study of 70 patients. Laryngoscope. 1999;109(6):949-953. doi:10.1097/00005537-199906000-00021 10369289

[25] Thomson PJ, Wylie J Interventional laser surgery: an effective surgical and diagnostic tool in oral precancer management. Int J Oral Maxillofac Surg. 2002;31(2):145-153. doi:10.1054/ijom.2001.0189 12102411

