# Supplementary material for: Effect of Fluorescence Visualization–Guided Surgery on Local Recurrence of Oral Squamous Cell Carcinoma: A Randomized Clinical Trial
Source: JAMA Otolaryngol Head Neck Surg. 2020 Oct 8;146(12):1149–55. doi: 10.1001/jamaoto.2020.3147 (PMC7545352; doi:10.1001/jamaoto.2020.3147)
Supplement: Supplement 1. — Trial Protocol [file jamaotolaryngolheadnecksurg-e203147-s001.pdf]

J. Scott Durham, Penelope Brasher, Donald W. Anderson, John Yoo, Rob Hart, Joseph C. Dort, Hadi Seikaly, Paul Kerr, Miriam P. Rosin, Catherine F. Poh. **Fluorescence Visualization with VELScope on Localized Oral Cancer Margins Does Not Improve Local Control: A Randomized Controlled Trial**

eMethod. Trial Protocol

eMethod. Trial Protocol

Fluorescence Visualization with VELScope on Localized Oral Cancer Margins Does Not Improve Local Control: A Randomized Controlled Trial

Authors: J. Scott Durham,[1] Penelope Brasher,[2] Donald W. Anderson,[1] John Yoo,[3] Rob Hart,[4] Joseph C. Dort,[4] Hadi Seikaly,[5] Paul Kerr,[6] Miriam P. Rosin,[7] Catherine F. Poh[8]

Affiliations

[1]Department of Surgery, Faculty of Medicine, University of British Columbia, Vancouver, BC, Canada

[2]Department of Statistics, Faculty of Science, University of British Columbia, Vancouver, BC, Canada

[3]Department of Otolaryngology – Head and Neck Surgery and Oncology, London Health Sciences Center, London, Ontario, Canada

[4]Division of Otolaryngology – Head and Neck Surgery, University of Calgary, Calgary, AB, Canada

[5]Department of Surgery, Division of Otolaryngology, Faculty of Medicine and Dentistry, University of Alberta, Edmonton, AB, Canada

[6]Deparmtent of Otolaryngology – Head & Neck Surgery, Rady Faculty of Health Sciences, University of Manitoba, Winnipeg, MB, Canada

[7]School of Biomedical Physiology and Kinesiology, Simon Fraser University, Burnaby, BC, Canada

[8]Department of Oral Biological and Medical Sciences, Faculty of Dentistry, University of British Columbia, Vancouver, BC

Funding Agency: Terry Fox Research Institute

Project Number: 2009-24

ClinicaTrials.gov Identifier: NCT01039298

TABLE OF CONTENTS

A.	STUDY OVERVIEW	4
B.	STUDY BACKGROUND	4
C.	PATIENT SELECTION	7
D.	ENDPOINT EVALUATION	8
E.	STATISTICAL CONSIDERATIONS	
F.	STUDY SITE ESTABLISHMENT	12
G.	QUALITY CONTROL	12
H.	PROJECT TEAM	14
I.	STUDY SITE PROTOCOL	16

Appendix 1. Eligibility Checklist for Randomization
Appendix 2. Patient Demographic Sheet
Appendix 3. Addendum Information Sheet
Appendix 4. EQ-5D, FACT-H&N, VHI-10 Health Questionnaire
Appendix 5. Lesion Tracking Sheet
Appendix 6. Surgery and Pathology Tracking Sheet
Appendix 7. Pathology Synoptic Report Form
Appendix 8. Surgery Resource Utilization Form
Appendix 9. Patient Information Update Form
Appendix 10. Biopsy Tracking Sheet
Appendix 11. Indirect Patient Cost Questionnaire (Baseline / Follow-up)
Appendix 12. Serious Adverse Event Report
Appendix 13. Adverse Event Log
Appendix 14. Speech Handicap Index (SHI)

A. STUDY OVERVIEW

Objective: This study is designed to assess the effect of FV-guided surgery on the local recurrence-free survival (L-RFS) of histologically confirmed disease within the context of a multicenter, randomized controlled trial.

Rationale: A single center longitudinal study has shown a significant impact of FV on local recurrence. The goal is to demonstrate similar results in a multicenter pan-Canadian study.

Study Design: The overall schema for the study is shown in **Figure 1**. A detailed step-by-step procedure is described in Section I. Study Site Protocol.

Figure 1. Study schema

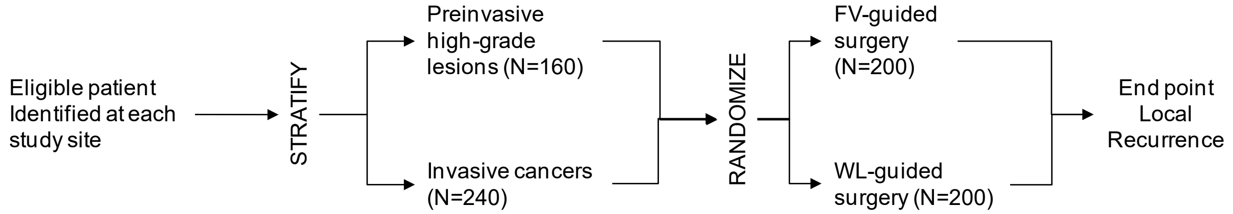

Study endpoints:

1. **Primary endpoint**
   Local recurrence-free survival
2. **Secondary endpoints**
   2.1. Failed first-pass surgical margin
   2.2. Regional or distant metastasis
   2.3. Death due to disease
   2.4. Quality of Life (QoL) measures

B. STUDY BACKGROUND

Oral squamous cell carcinoma (SCC) is a global disease responsible for ~300,000 new cancer cases each year.[1] It is believed to progress from oral premalignant lesions (OPLs) to invasive squamous cell carcinoma (SCC). Once cancer has developed, it has one of the worst prognoses of epithelial cancers, with 5-year survival rates ranging from 30- 60%, depending on the global locale. Local recurrence is common, present in up to 30% of cases.[2,3] Cosmetic and/or functional compromise associated with treatment of disease is often significant. These statistics underscore the urgent need to develop new approaches to better control this deadly disease. There has been extensive research on the importance of examining the field surrounding oral cancers for both risk assessment and management of this disease.[4] Using molecular technology, it is becoming increasingly apparent that genetically altered cells are often widespread across the mucosa of patients with oral cancer, extending into clinically and histologically normal tissue, and that these cells can drive the process of field cancerization.[5,6] In recognition of this, surgeons try to remove oral squamous cell carcinomas (SCC) with a significant width of surrounding normal-looking oral mucosa (usually around 10-mm). However, the occult disease varies in size and a wealth of evidence suggests that it frequently extends beyond the tumor clearance area. This extension may be responsible for the high rate of cancer recurrence at the primary site

(10-30% of cases).[7-9] Since occult disease varies in size, this approach can result in either over-cutting (causing severe cosmetic and functional morbidity) or under removal of disease tissue, as evidenced by frequent positive surgical margins and high local and regional recurrence – a failure of the 'best practice'.

Direct Fluorescence Visualization (FV): an adjunct tool for facilitating clinical identification of high-risk tissue. There is a wealth of literature that supports the use of tissue autofluorescence in the screening and diagnosis of precancers in the lung, uterine cervix, skin and oral cavity.[10-16] This approach is already in clinical use in the lung 35 and the mechanism of action of tissue autofluorescence has been well characterized in the cervix.[17-19]

Changes in fluorescence reflect a complex interplay of alterations to fluorophores in the tissue and structural changes in tissue morphology. These changes have been associated with progression of the disease.[19,20] Autofluorescence originates from endogenous fluorophores in the oral mucosa. Important fluorophores in the epithelial layer include the metabolic co-factors NADH and FAD while cross-links of the collagen, a structural protein, are the principle fluorophores of the lamina propria.[20,21] The intensity and wavelength of the autofluorescence provides information about the tissue's local biochemical composition, its structure (i.e. epithelial thickness), and metabolic activity, which are intimately related to the disease state. Alterations to fluorophore distribution include tissue remodeling such as the breakdown of the collagen matrix and elastin composition as well as alterations to metabolism.[22] Structural changes in tissue (e.g. thickening of the epithelium, hyperchromatism and increased cellular/nuclear pleomorphism, or increased micro-vascularity) lead to altered absorption and/or scattering of light which in turn reduces and modifies the detectable autofluorescence signal.

Several studies have shown that spectroscopy of autofluorescence can discriminate between normal and neoplastic mucosa.[11,14,21,23-26] In a more recent study, Svistun *et al*.[27] recorded autofluorescence from freshly resected oral tissue at specific excitation and emission wavelengths. The best results were achieved with illumination at 400 nm and observation at 530 nm. When tumor margins determined from direct FV were correlated with Histopathological diagnosis, a sensitivity and specificity of 91% and 86% respectively were achieved in discriminating normal tissue from neoplasia. However, that study was conducted with *ex vivo* tissue samples and the bulky machinery setup limited its clinical application. We have recently developed a simple hand-held field-of-view device for direct visualization of tissue fluorescence in the oral cavity. This FV device, currently marketed as VELScope®, has been used to follow clinical changes to the oral mucosa of all patients in our Oral Cancer Prediction Longitudinal (OCPL) study (funded by NIDCR/NIH since 2004).[28] As shown in Figure 2, the FV device has demonstrated an ability to detect a new, expanded definition of the altered field, that could impact significantly on disease management. We are now using the device in the operating room to directly visualize subclinical field changes around oral cancers.[29,30] Early findings with this approach are exciting and promising.

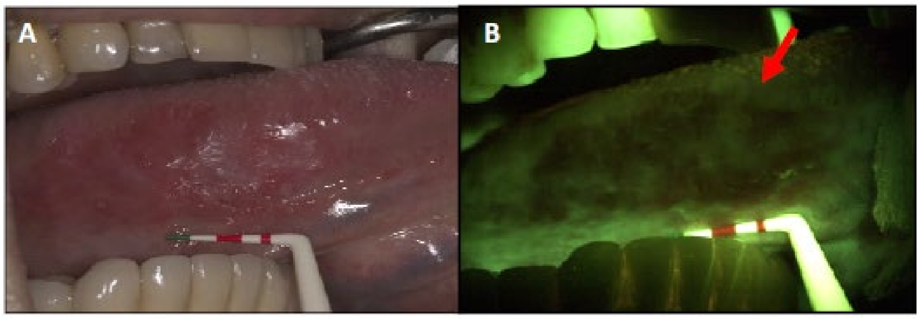

Figure 2. Assessment of a lesion at right side of tongue using white light (WL) (A) showing an ill-defined lesions; using FV in the dark (B) showing a well-defined area of dark / dark brown change (red arrow)

Previous work
British Columbia is unique in the world in that it has centralized much of the oral cancer/precancer control processes in the province within an oral cancer prevention program that links cases detected in dental screening networks through a province-wide oral biopsy service to dysplasia referral clinics for assessment, with flow into hospital facilities for treatment and back for follow-up. This referral system has enabled the creation and development of an unique longitudinal study (OCPL study) that has as its goal the evaluation and testing of clinical and molecular technology for risk assessment (ability to predict progression to cancer, local recurrence of disease, and second primaries).[31,32] This existing framework has enabled the very rapid development and commercialization of the novel FV technology being used in this study.[28] This technology, the VELScope® has Health Canada and FDA approvals as an adjunct to white light screening and based upon the high-risk margin data presented above also has approval for determination of surgical margins. The patients on which this FV-enabled alteration in clinical practice was performed have been followed in BC over the last 3 years. Specifically the linkage to the OCPL study has allowed collection of outcome data of patients for which this clinical practice change was applied.[4]

Pre-invasive high-grade lesions (HGLs) and treatment
High- grade lesions (HGLs, severe dysplasia/CIS) are often characterized by persistence, recurrence and eventual progression to invasive SCC.[33] There is no consensus on how such lesions are managed. Quite often, they are treated with surgery, although no guidelines exist on how much normal margin should be removed at resection if any. On the other hand, clinicians may elect to monitor the lesion for progression rather than treating it. This decision is largely based on the clinician's perception of how much risk the lesion has of progressing and a hesitancy to "over treat" if the lesions should prove to be benign in behavior. Of interest, the frequent recurrence that often follows treatment of such lesions implies incomplete excision, possibly due to the presence of subclinical change at the margins that is not removed.

In BC, until recently, decision to treat HGLs was left with the clinicians. However, 3 years ago this practice changed to a recommendation of surgery for such lesions. This change was a direct consequence of data obtained from the ongoing longitudinal study on progression rates for HGLs. In that analysis, a total of 124 HGLs were identified, some found in cancer patients, others in primary lesions. Eighty (65%) of these lesions had been treated by surgical excision, while the remaining lesions were left for follow-up. For patients not receiving treatment, progression rates to cancer were 42%, 56% and 70% in 2, 3, and 5 years of follow up, respectively. Progression rates were reduced in patients receiving surgery. This high progression

rate clearly supports the need for treatment of HGLs. Furthermore, the frequent recurrence following excision for such lesions suggests that, like SCCs, the presence of subclinical change the margins not apparent at surgery might result in incomplete excision.

We have recently reported pilot data on 22 HGLs that were treated using FV-guided surgery. FVL was apparent beyond clinical boundaries in 21 of these lesions. Similar to previous observations in SCCs, the subclinical extension of FVL around these HGLs was uneven, ranging from 1 to 25 mm in width with extensions similar to those observed with SCC. Strikingly, 35% (13/37) of biopsies from the outer FV boundary of HGL showed histologically high-grade change; in 5 biopsies this change occurred at beyond 10-mm, the conventional margin employed for cancer treatment. These data suggest that integration of FV in surgery might guide management of HGL at the point of care by identifying subclinical field change associated with high risk histology that should be treated aggressively. These data further support the importance of including HGLs in the present study to determine whether FV-guided surgery will impact on outcome for such lesions.

C. PATIENT SELECTION

a) Inclusion Criteria:

- Patients with high-grade preinvasive (severe dysplasia/ carcinoma *in situ*) or invasive squamous cell carcinoma (T1 or T2) of the oral cavity that will be undergoing curative resection for primary disease
- Disease localization at oral anatomical sites that can be visualized using both white light and fluorescence visualization device. This includes ICD-10 site codes: C02.0-C06.9[34]
- Clinical diagnosis of N0 or N1 as confirmed by CT scan, with the latter undergoing neck dissection
- Patients with resectable locally recurrent disease diagnosed with severe dysplasia and above, provided that they are at least 6 months post-treatment. This time frame will allow resolution of artefacts produced by treatment that could impact on tumour or lesion visualization
- Patients are willing to participate and to provide informed consent in the study

b) Exclusion Criteria

- Patients must not have a concurrent non-oral malignancy diagnosed within the past 3 years. Patients with non-melanoma skin cancer and lymphoma that lie outside of the head and neck region are included
- Patients must not have evidence of distant metastasis, as determined by CT and X-ray at the time of recruitment
- Patients must not have illness that could preclude standard diagnostic tests and postsurgery follow-up
- Patients with lesions located at the base of tongue (C01) or tonsil (C09) are excluded because these sites are not readily assessable to FV

D. ENDPOINT EVALUATION

1. **Primary endpoint:** Local recurrence – defined as recurrence at or within 1 cm of the surgical site, with the same or a higher grade histology than the initial diagnosis or further treatment due to the presence of severe dysplasia or higher degree of change at follow-up. Local recurrence-free survival is defined as the time from surgical procedure to local recurrence. Patients lost to follow-up due to moving, refusal or death from other causes will be censored at the time of last follow-up.

2. **Secondary endpoints:**
   - 2.1. **Failed first-pass margins:** Presence of histologically confirmed margin for severe dysplasia or greater histgological changes
   - 2.2. **Regional or distant metastasis:** At any follow-up time point, failure of regional or distant control, i.e., development of metastatic disease to regional lymph nodes confirmed by fine needle aspiration, CT or MRI, or subsequent pathology diagnosis. Patient's death due to disease recurrence, including failure in local, regional and distant control, is considered an event. Patients who die of unrelated events such as breast cancer, prostate cancer, cardiovascular disease, or other unrelated causes, are censored at the time of event.
   - 2.3. **Death due to disease:** Patient's death due to advanced, recurrence, or metastasis from primary oral cancer.
   - 2.4. **Quality of Life (QoL) measures.** To assess potential psychosocial consequences of surgery, we will use the EQ-5D[35,36] and Functional Assessment of Cancer Therapy Head and Neck Module[37,38] (FACTH&N) to measure global QoL to determine the participant's QoL at each assessment: at presurgery baseline, at 6-week, 3-month, and 24-month post-surgery follow-ups. A specific tool for the measurement of speech pathology (the Speech Handicap Index)[39] will also be used to measure the specific impact that treatment in either arm has on patient speech performance.

E. STATISTICAL CONSIDERATIONS

The proposed study will be a double-blinded, controlled randomized Phase III study to evaluate the effect of FV-guided surgery in patients diagnosed with severe dysplasia, carcinoma *in situ* and invasive squamous cell carcinoma and undergoing surgery treatment with intent-to-cure. We plan to recruit a total of 400 patients in the first 3 years with at least an additional 2 years of follow-up. The total study period will be 5 years. Interim analyses will be conducted at approximately at the end of Year 3 and Year 4 and the final analysis at the end of Year 5. Patients will be randomized into the FV-guided surgery and conventional white light surgery (without FV guidance) with equal probability. We expect a small percentage of early drop out or loss to follow-up (up to 10%) due to the nature of higher compliance of the elevated risk groups, a well established clinical trial infrastructure, and intense follow up in this population.

The primary endpoint of the local recurrence-free survival (LRFS) defined as time from surgical procedure to the development of histologically confirmed disease recurrence including the development of new lesions with severe dysplasia, carcinoma *in situ*, or invasive squamous cell carcinoma or death, whichever occurs first. To qualify for recurrence, the post-surgical histology must be at least as severe as the baseline histology. For example, for patients with severe dysplasia, a new lesion developed with severe dysplasia or worse histology is considered as

recurrence while, for cancer patients, only the development of new cancer is considered as recurrence. Due to the intensive follow-up schedule, early identification of recurrent disease can be achieved. All patients will be analyzed on an intent-to-treat basis (i.e., as randomized). Patients lost to follow-up due to moving, refusal or death from other causes (if clearly documented) will be censored at the time of last follow-up.

The distribution of LRFS will be estimated by the Kaplan-Meier method. For randomization purposes, patients will be stratified by institution and stage of preinvasive high-grade lesion (HGL, severe dysplasia and carcinoma *in situ*) or invasive squamous cell carcinoma. Within each stratum, Pocock-Simon dynamic allocation method will be applied to achieve balanced randomization with respect to other factors, including surgeon, gender, age, smoking history, and lesion anatomical sites. Stratified log rank test will be used to compare LRFS among groups. The Cox (proportional hazards) regression model will be used to incorporate potential prognostic factors and the treatment assignment as covariates. Details of the assumption used for the sample size calculation are listed below.

1. Based on our preliminary data acquired from British Columbia and Ontario, we observe that the 3-year recurrence rate in this patient population can be as high as 50% for HGL patients and 30% for cancer patients (Figs. 1 and 2). Taking a conservative estimate, for the sample size calculation, we assume that the LRFS follows an exponential distribution with a 3-year recurrence rate of 40% for the HGL patients and 25% for the cancer patients. These two rates will be used as the event rates in the control (white light surgery without FV guidance) groups.
2. Our preliminary results show that FV is highly effective with a 3-year recurrence rate of 10% for the HGL patients and 5% or less for the cancer patients. We expect that there will be a learning curve for FV training. To be conservative, it is also safer to assume a smaller treatment effect when the technique is applied to general population in different provinces and different surgeons in different medical institutions. Therefore, we assume that the FV treatment can reduced the 3-year recurrence rate from 40% to 20% in the HGL patients and from 25% to 11.5% in the cancer patients. The assumption corresponds to a hazard ratio of 0.43 for the FV-guided surgery versus non-FV-guided surgery, with 57% reduction in the event rate.
3. Stratified randomization will be performed. Two stratification factors will be applied: (a) 8 major institutions participate in this multi-center trial and (b) histology of the primary lesion (HGL or invasive carcinoma). Our goal is to enroll 40% HGL patients and 60% cancer patients. Stratified log-rank test and Cox regression model will be used for testing the effect of FV approach.
4. The results may be different from surgeon to surgeon participating in the trial. Effects of surgeons can be considered as random effects.
5. Group sequential test will be applied with when approximate one-third and two-thirds of predicted outcomes have occurred (estimated to be at the end of year 3 and year 4) with a final analysis at the end of Year 5. O-Brien-Fleming's stopping boundaries will be applied. The corresponding P values for declaring significant results at Years 3, 4, and 5 are 0.0005, 0.014, and 0.045, respectively. The study result will be reported to the Data Safety and Monitoring Board on a yearly basis. If the study confirms the effectiveness of FV and the

early results cross stopping boundaries, the trial will be stopped early. FV can be adopted as the standard of care.

6. We assume up to 10% of patients will be lost to follow up and be considered as censored.
7. Simulation studies were conducted with 1,000 runs. Based on the above assumptions, to reach at least 85% power with an overall, two-sided 5%significance level, a total of 400 patients will need to be enrolled in three years and followed for additional two years. The projected total study duration is 5years. We will also achieve at least 70% power in the subgroup of 160 HGL patients and the subgroup of 240 cancer patients.

Figure 3. Time to recurrence to the same or worse histology in patients with high-grade lesions (HGL: high-grade lesions include severe dysplasia or carcinoma in situ). FV, fluorescence visualization. Early and CNTL are patients without FV from early and recent cohorts.

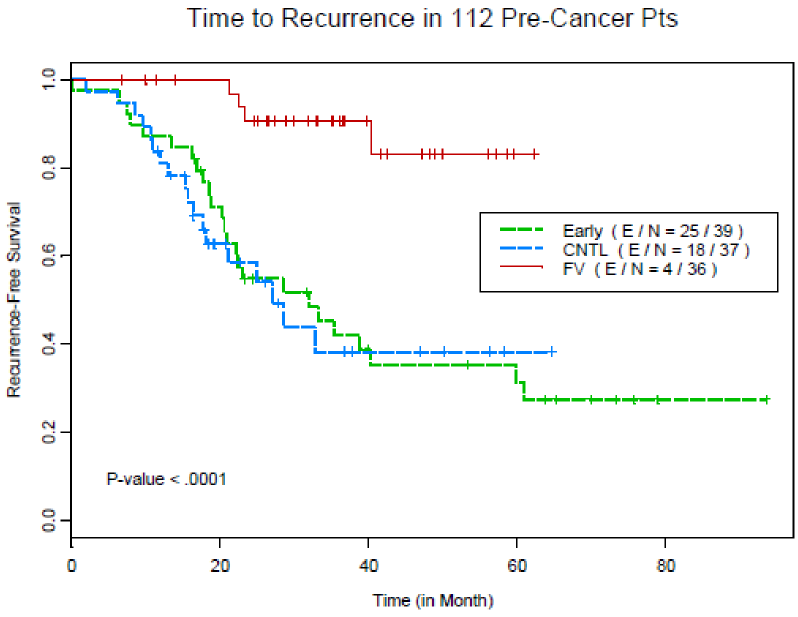

Figure 4. Time to recurrence to cancer in patients with cancer

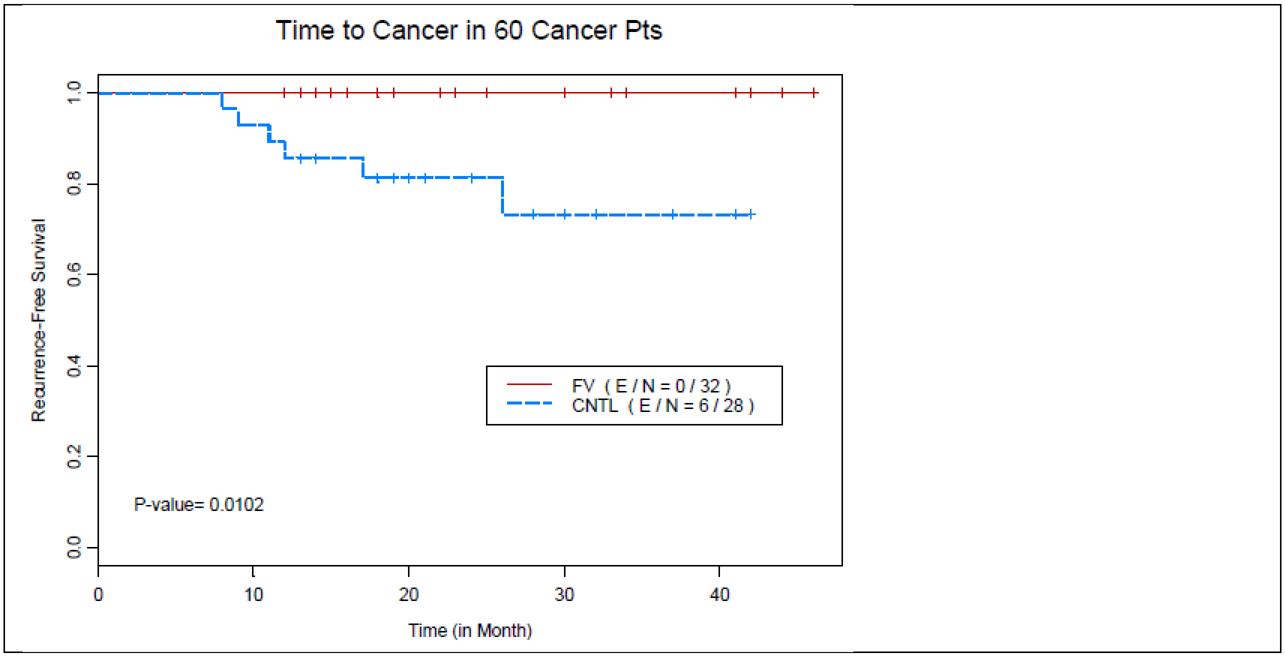

Time to Cancer in 60 Cancer Pts
Recurrence-Free Survival
1.0
0.8
0.6
0.4
0.2
0.0
0
10
20
30
40
Time (in Month)
FV (E / N = 0 / 32)
CNTL (E / N = 6 / 28)
P-value= 0.0102

F. STUDY SITE ESTABLISHMENT

Much of the early activity of this project will be focused on transferring Vancouver expertise to the seven other study sites. It will be necessary to establish uniform standards of practice (SOPs) for all key activities; however, each site will have its own unique environment and personnel. A fine-tuning of the protocol will occur during the initial site visits to accommodate these differences. During these visits, the training of the multidisciplinary team of surgeons, pathologists, project coordinators, and local FV Specialists at each site will occur.

Investigators in Central Management have over 6 years of experience with FV and over 10 years experience in the numerous aspects required to successfully run long-term clinical studies, including patient accrual, recruitment and retention, which are key to the success of the proposed study. Initial visits to the study sites by members of central management will provide us with an understanding of each site's unique characteristics and will help us to work with each site to ensure that the protocols will run effectively. It will provide us with an opportunity to provide hands-on training. Among the many components that will require attention are the following:

- Standardization of the steps used in performing a comprehensive head and neck and intraoral examination involving both white light and FV. This will involve training of site members in the use of the FV device, the *Velscope* (Site Surgeons, FV Specialist and, Site Coordinators);
- Use of the Specialized Fluorescence Flash Camera for taking the high quality FV and white light images critical to the study (Site Surgeons, FV specialists, Site Coordinators);
- The marking of clinical and fluorescence boundaries and use of these boundaries to determine the surgery boundary, with documentation of these processes with digital images and with the study surgical tracking sheet (Site Surgeons, FHS personnel, Site Coordinators);
- Blocking of the tissue with study-specific margin notation and completion of the synoptic pathology form in the study e-database (Site Pathologists);
- Lessons learned on how to facilitate patient, recruitment, consenting and retention to study (Site Coordinators);
- Use of the scannable collection tools associated with this study, uploading of information from these forms, clinical information and images to the e-database (**Site Coordinators**); and Standards of practice for handling biospecimens, including packing and shipping of the archival and fresh frozen samples (**Site Coordinators**).

G. QUALITY CONTROL

The acquisition of high quality data is the key to the success of any clinical trial. We have planned a systematic strategy for control of data and image capture with a special focus on ensuring that the involved processes are efficient and of high-quality. The main components of this strategy are described below.

1. Quality control of clinical data

The clinical research forms (CRFs) developed in BC for data capture during the ongoing longitudinal study will be fine-tuned and then translated into a set of scannable teleforms for use

across sites. These scannable forms can be quickly uploaded to an e-database that will be created for this study. The use of scannable forms will significantly increase the efficiency of the knowledge capture and avoid errors during the data collection process. An email alert will be built into the database that will be activated when key data is uploaded to this database, such as information needed for eligibility assessment of a patient and randomization. The Centre Program Manager (CPM) and the Administrative Assistant (AA) will receive this email and can confirm accuracy. The AA will perform a day-to-day check to ensure the completeness of data as it is uploaded, identifying missing information quickly and dealing with it through communication with the Site Coordinator. The CPM and AA will work together to ensure that all data captured are complete and accurate.

2. **Quality control of clinical images**

During the training period, all FV operators (FVS and site surgeons) will need to pass a 2-step control process: **Step 1.** As part of the training process, a set of 10 images will be provided to the site surgeons and FVS, as a first step in calibrating their judgment on clinically visible tumor boundaries and FV positive (FV loss) boundaries. The criteria for passing the step is that both clinically visible tumor boundaries and FV boundaries need to be within ± 5 mm of those drawn by the experienced FV operators from the BC site. **Step 2.** To be certified, all FV operators require hands-on experience in outlining the tumor and FV boundaries on **3** real patients. The same criteria will be used to assess this activity, with a requirement that both clinically visible tumor and FV boundaries be within ± 5mm of those drawn by the experienced FV operators from BC site. This time, the BC specialists will draw on the images from the study sites, since they are not likely to be present during the actual surgeries. After passing this 2-step control process, this site will be ready to recruit the first patient to the study.

During the study period, Dr. Poh will work with the AA to review the first 10 patients from each site to ensure the quality of images is maintained and that mapping of clinical and fluorescence boundaries proceeds as per initial training and to provide suggestions to the site team members for any problems experienced during these activities. Through this reviewing process, the AA will be trained to identify good quality white light and FV images. This will enable the AA to continue the process of reviewing all images uploaded from each site on a daily basis as an early indicator of problems. The AA will report to the CPM and Dr. Poh if the quality of images is poor. In addition, Dr. Poh and AA will do a random check of incoming images to ensure the continuation of high-quality image documentation.

3. **Quality control of operation**

Three complete cases using FV-guided surgery must be reviewed in order for the site to be qualified for patient recruitment. In addition to the regular data and image monitoring that will occur throughout the study (described above), the CPM will periodically perform a random check of all processes of selected cases, with at least 1 case from each site every 3 months to ensure the continuation of high-quality operation and integrity of the study. The computer database will be set-up to alert the Centre and Site if a patient is overdue for their scheduled follow-up appointment. A bi-weekly to monthly teleconference will share problems among sites. Any operational questions in the project will be brought forward to the Centre Management Committee. The CPM and AA will work towards a solution depending on the input from the Committee and all problems and solutions will be logged for future reference. Annual travel for

site visit will further ensure the integrity of the entire operation and assist in site trouble shooting at each study site.

H. PROJECT TEAM

The **Central Management** includes the Centre Management Committee (for day-to-day management and quality assurance of the project-related issues), the Database and IT Committee (for database and IT related issues), the Steering Committee (for oversight of the project), and the Outcome Jurisdiction Committee (for Endpoints). In addition, we will have an External Advisory Scientific Committee (for scientific advice and guidance to the project) and a Data Safety and Monitoring Board (will be constituted to independently monitor the project).

Site Management: Each site will have a working team comprised of the Site Coordinator, Site Surgeon(s), Site Pathologist and FV Specialist. The responsibilities of these personnel are presented in Appendices 5 and 9. Site members will liaise with central management for day-today operation. This will be coordinated through interactions of the Site Coordinator and the Center Management Committee. Site team members can forward concerns to that committee through the Site Coordinator. The Lead Site Surgeon, if there is more than one surgeon, will work with and supervise the site coordinator for site specific patient accrual and data collection and maintain working relationship with SP and FVS.

Center Management Committee, CMC (Quality Assurance, QA): This committee will provide day-to-day oversight of activities across all sites with a primary focus on quality control of study protocols and timely completion of milestones. Membership will include: the Central Program Manager and Center Administrative Assistant as well as the Site Coordinators from each site. This committee will have regular access to Drs. Rosin, Poh, and Durham through the Center Program Manager. Dr. Rosin will have oversight on issues surrounding overall management and sub-project coordination. Dr. Poh will provide input on pathology, FV assessment and data and sample collection. Dr. Durham will provide input for issues around surgery. The CMC will have regular weekly meetings through web-based interfaces (i.e., Skype) and/or teleconference if it is involve with all or multiple sites with an additional open communication at any point in time through both email and telephone.

Histology Central Review Committee (QA): This committee will confirm histological diagnoses of surgery and follow-up biopsies. Key to this activity will be the centralized scanning of H&E specimens to allow simultaneous review across centres. This committee will include Dr. Ken Berean (Vancouver) and Dr. Martin Bullock (Halifax). Any discrepancy between the histological diagnosis from the study sites and the central review will be dealt with through either email and/or teleconference with Pathologist(s) at that site. A critical component of this committee will be the confirmation of histological diagnosis at study entry in order to ensure that cases are eligible, i.e., have a diagnosis of severe dysplasia, *CIS* or SCC. This review is also critical for confirmation of endpoints. This data will flow forward to the Outcome Jurisdiction Committee.

Database and IT Committee (QA): The Database/IT Committee will deal with data and IT-related issues. It will involve contracted IT-support under the supervision of Drs. MacAulay

and Brasher. Site Coordinators will access resources of the centralized Database/IT Committee through contact with either the Center Program Manager or the Administrative Assistant.

Steering Committee: The steering Committee will provide oversight to the clinical trial and each of the Subprojects. Membership on the committee will include: Dr. Miriam Rosin (Project Director), Dr. Scott Durham (Project Co-Director), Dr. Catherine Poh (Project Co-Director), Dr. Calum MacAulay (Project Imaging Consultant and Co-Leader for Subproject 3), Dr. Stuart Peacock (Leader for Subproject 2), Dr. Kitty Corbett (Leader for Subproject 4), Dr. Penny Brasher(Project Biostatistician), Dr. Ken Berean (Project Pathologist), Dr. Joe Dort (Project Head/Neck Surgical Oncologist), Dr. Jack Lee (Study Design Consultant) and the Central Program Manager. This committee will have regular monthly meetings.

Outcome Jurisdiction Committee: Will provide an unbiased evaluation of outcome, looking at clinical and histological data for use by the Data Safety and Monitoring Board in their review process. Membership not yet complete, but will include: Dr. Penny Brasher (Biostatistician, representative from Steering committee), Dr. John Hay (Radiation Oncologist, tentative), and Dr. Kenneth Berean, a representative from the Histology Central Review Committee.

Scientific Advisory Board: Will involve individuals nationally and internationally known for work with clinical trials, study endpoints in this project and/or with cancer control strategies. This committee will receive input from both the Steering Committee on a quarterly basis and will meet annually to provide advice to the steering committee on study design aspects. This committee is still in development: Drs. Andy Coldman (Provincial Leader for Population &Preventive Oncology, BC Cancer Agency, specializing in cancer prevention and population health), Mark Elwood (Vice President for Family & Community Oncology, BC Cancer Agency, specializing epidemiologist and public health medicine), and Jack Lee (Division Chair of Quantitative Sciences, Department of Biostatistics and a Kenedy Foundation Chair in Cancer Research at the University of Texas M. D. Anderson Cancer Center, Houston, Texas, specializing in clinical trial study design).

Data Safety and Monitoring Board (DSMB): Yet to be appointed. This committee will independently evaluate study progress at regular intervals, looking at safety, data and pathology quality and efficacy of the device. The board will meet at the beginning of the trial, and at the time of one third and two third of the expected number of patients to reach the endpoints (local recurrence, regional and distant metastasis). Membership includes: Chair (Dr. Stephen Chia, a medical oncologist who is experienced in clinical trials, Head and Neck Oncology, and DSMB issues), a surgeon (to be appointed), a head and neck radiation oncologist (Dr. John Hay, immediate past chair of the Head and Neck Tumor Group, BC Cancer Agency), and a biostatistician (Dr. Dong Sheng Tu, Biostatistician in NCIC-CTG, Kingston).

I. STUDY SITE PROTOCOL

A schematic delineating workflow is presented below in Figure 5. The following abbreviations are used for personnel: **SS**, Study Surgeon; **SC**, Site Coordinator; **FVS**, FV Specialist; **SP**, Study Pathologist. In order to keep the study double blinded and avoid potential bias during follow-up, the FVS will be a different person from the SS.

Figure 5. Workflow chart depicting key steps of the protocol and responsible study staff, including clinicians (SS, SP, FVS) and site coordinators (SCs)

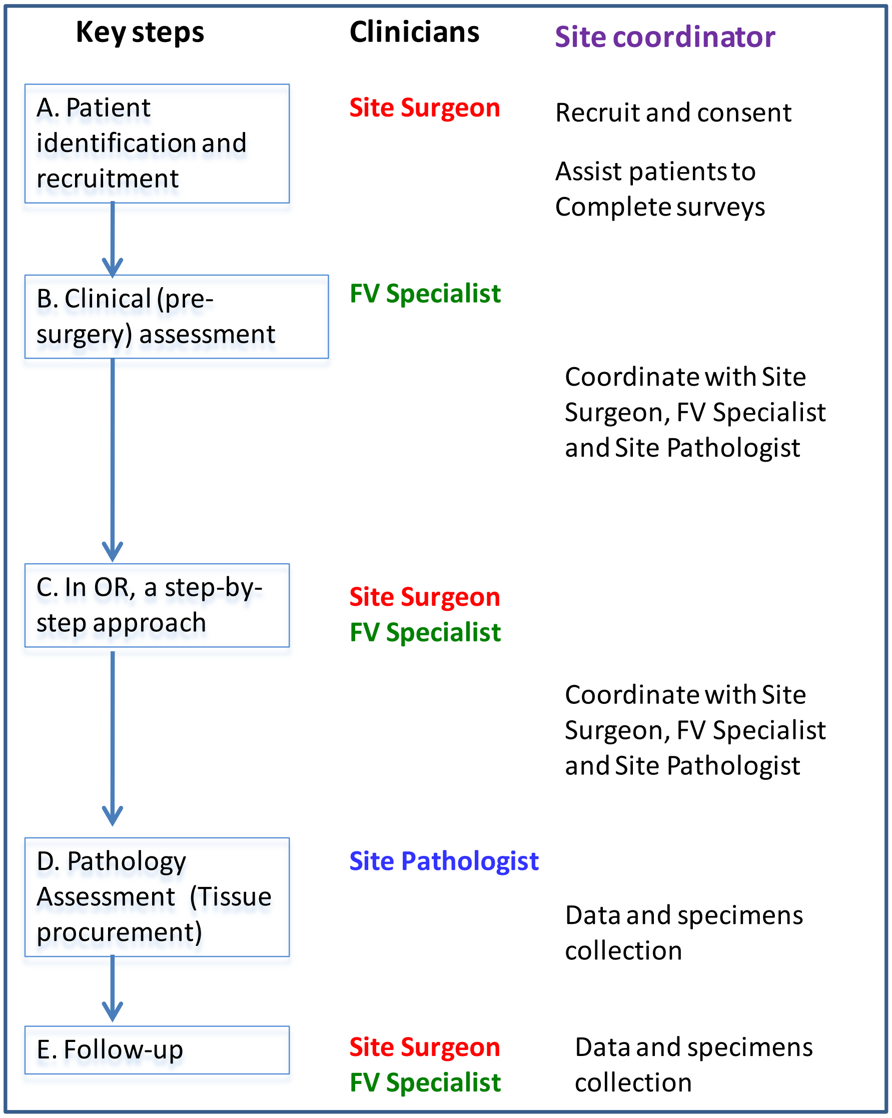

1.0 **Registration Procedures**

1.1 The SS will identify potentially eligible patients and inform the SC. SC will use the eligibility check list to confirm patient's eligibility

NOTE: All eligible patients should have a CT scan from skull base to chest as a baseline to confirm the nodal status and the absence of the Upper Alimentary and Respiratory Tract (UART) and Lung metastasis or Second Primary Tumor.

2.0 **Pre-surgery Assessment (at FVS's office)**

2.1. The SC will introduce the study and obtain informed consent from the patient. NOTE: Consent must be obtained prior to surgery.

2.2. Completion of surveys for demographics, comorbidity and quality of life (generic and disease specific) by patient with the assistance of the SC.

2.3. WL assessment (SS and FVS): SS and FVS will assess the clinical lesion together or independently. FVS will take image and measure the size of the lesion and SC will help to put it down on the grid sheet.

2.4. FV assessment: FVS will assess the lesion under FV and take images without the presence of SS. This is a necessary step to keep SS 'blind'.

NOTE: At this time the lesion undergoing treatment will be visualized using white light (WL) and fluorescence visualization (FV). Digital images using the Specialized Fluorescence Flash Camera System for documentation of WL and FV status will be obtained.

3.0 Randomization (On study homepage, the SC will key in the required information and assign the patient group by the randomization program provided).

4.0 Surgery (In operating theatre and patient under general anesthesia)

NOTE: The SC will find out the OR booking and coordinate among SS, SP, and FVS, prepare and deliver the surgical package (tracking sheet and pens) to FVS and the pathology package (tracking sheet and Paroloid camera [optional: tissue moulds, dry ice for tumor banking; we will work out the logistics for this with each SP individually]) to the pathologist, and pick up both packages for data entry after procedure.

NOTE: All the assessments (WL and FV) are prior to local anesthesia. If sutures are used to immobile the surgical area, please place them in a way that will not interfere with the visualization and assessment.

NOTE: 2 6 ml EDTA tubes and 1 6ml SSP tube of blood sample can be collected at this time prior to the procedure (see SOP of the blood sample collection for details).

4.1. SS will outline the boundary of the clinically visible lesion under white light. The FVS will take an image (**Image 1**), measure its size, and record data on the surgical tracking sheet.

4.2. **If this is a FV surgery**: With the OR light off, the FVS will use the FV tool to take an image (**Image 2**) under FV in the dark to demonstrate the distance between

FVL and clinical outline. The FVS will outline the FVL boundary under FV on the oral mucosa. With the OR light back on, the FVS will take another image (**Image 3**), measure the distance from FV to the clinical boundary in 4 directions, and record this on the surgical tracking sheet.

The SS will outline a 10-mm surgical boundary around the clinical AND FV boundaries whichever is wider. The FVS will take images under both FV and WL (**Image 4 & 5**) and record if there is any anatomical restriction for the placement of the standardized surgical boundary.

If this is a WL surgery: After step 4.1, the OR light will be turned off and the FVS will use the FV tool to take an image (**Image 2**) under FV in the dark to demonstrate the distance between FVL and clinical tumour outline. After the imaging, FVS will outline only on top of the surgeon's clinical boundary (this step is necessary to keep the surgeon blinded). The OR light will be turned on, and the FVS will take another image (**Image 3**). In this case, the 2 outlines will be identical and this will be recorded on the surgical tracking sheet.

The SS will outline a 10-mm surgical boundary around the clinical/FV boundary. The FVS will take images under both FV and WL (**Image 4 & 5**) and record if there is any anatomical restriction for the placement of the standardized surgical boundary.

4.3. FVS will complete the surgery tracking sheet with margin information.

4.4. The tumor will then be resected and oriented using a suture for the anterior or right orientation. This can be indicated on the routine pathology requisition form to help the SP to orient the resected tissue. The specimen will be wrapped in a piece of cold saline gauze (preferably on ice) and delivered to the Pathology Department for processing. The FVS will page the SP.

5.0 Specimen processing (In the gross room of the Pathology Department)

5.1. The pathologist will pin the tissue to a piece of wax waffle and take a picture using a Polaroid camera/film and make sure the outlines are clear.

5.2. A portion of tumor tissue will immediately be harvested (preferably 5 x 5 mm in dimension) and put into the cryomold with OCT in a dry ice container. Some of the sites might be able to obtain margin fresh frozen tissue. The location of the fresh sample will be marker onto the Polaroid image. The SC will be paged to pick up the frozen tissue sample.

NOTE: This sample will only be taken if it does not hinder routine diagnosis and standard histopatholoigcal assessment of the margins.

5.3. The tissue and wax waffle will be placed into 10% neutral formalin overnight.

5.4. The tissue will be blocked and blocking recorded on the Polaroid image of the specimen. To better demonstrate the margins at 4 directions, the specimen can be blocked as follows: Perpendicular sectioning (parallel to the coronal section perpendicular to the anterior-posterior direction) of the clinical tumor area to demonstrate the superior and inferior surgical margins; horizontal sectioning of the remainders (anterior and posterior portions) to demonstrate anterior and posterior surgical margins.

5.5. The SP will put down key fields in the database through a Pathology Synaptic Format on the web-based database interface.

6.0 Patient Follow up

Note: The SC will coordinate the booking for patients' follow-up after surgery. This can be done at either the surgeon's or FVS's office.

6.1. **Schedule of follow-up**: at 6 weeks, 3 months and then every 3 months until the 2 year post operative anniversary then every 6 months for the remainder of the study period.

6.2. Survey data collection: quality of life surveys and updated risk factor survey (at 6-week, 3-months, 6months and 2-year post surgery).

6.3. Clinical data collection: lesion tracking sheet and images for each follow-up visit.

- WL assessment (FVS and/or SS): Examination of entire oral mucosa under WL and with collection of WL images for surgical site and other sites of interest. If there is any clinically visible lesion, with the assistance of SC, will record the clinical size and location on the tracking sheet. If not, note condition of scar or graft on the tracking sheet.
- (Optional) FV assessment (FVS and/or SS): Examination of entire oral mucosa under FV with FV images for the surgical site and wherever there are FV findings. Also retake WL images at such sites, if this has not yet been done during the WL examination. A record of the size of FVL at the time of examination, even it is a scar under WL, will be put onto the tracking sheet.

6.4. Imaging: Repeat CT scan, if there is clinical suspicion of regional or distant diseases. If there is no clinical indication, Neck CT scan and a chest X-ray will be arranged at 2-year post surgery follow-up.

6.5. Timing for biopsy during follow up. This will depend on the clinical judgment of the surgeon (SC should be informed if this is done by FVS). Usually it is at the time when the clinician suspects a recurrence or at the time of 2-year post surgery follow-up, if there is no significant clinical or FV finding.

NOTE: The indicator for significant FV findings is increase in size over 10 mm in one dimension.

Description of Site Personnel and Activities

Abbreviations: SS, Site Surgeon; GP, General Practitioners; ENT, Ear nose and throat specialist; SC, Site Coordinator; CMC, Central Management Committee; SP, Site Pathologist; FVS, Fluorescence Visualization Specialist.

1. Site Surgeons (SS):

One of the site surgeon will be the lead in the project. The SS is responsible for creating an active referral pipeline from GPs, ENT colleagues, dentists and radiation oncologists by use of various resources, e.g., conferences, meetings, or newsletters from local Colleges or Societies.

The SS will:

1.1 Identify eligible patients, make initial contact and facilitate recruitment. Each site must commit to identify and recruit **at least 40 eligible patients per year for the first 2 years.**

1.2 Arrange for initial staging investigation. All eligible patients should have a CT scan including the oral cavity, neck and chest. A CT scan will be required at 2 years follow-up.

1.3 Obtain all appropriate samples during the surgery (including blood sample collection).

1.4 Work with the FVS intraoperatively to maintain the integrity of the study and avoid contamination of the trial.

1.5 Maintain an ongoing working relationship with the FVS and SP.

1.6 Participate in patient follow-up and biopsy if necessary.

1.7 Some sites surgeons may perform follow-ups themselves, if they are interested in taking images. NOTE: The SS DOES NOT PERFORM PRESURGERY or SURGERY FV ASSESSMENT – this is done by the FVS (see 1.4. above).

1.8 Perform comparative biopsy at time of clinical change or at 2-year post surgery follow-up.

1.9 Assist SC to locate a -80 freezer for temporary storage of the frozen tissue prior to shipping.

1.10 Notify CMC for any study endpoints.

1.11 Be available to participate in teleconference for study quality assurance.

2. Site coordinator (SC):

This position represents a 0.5 FTE depending on qualifications (including benefit, up to 62K per year) for 5 years.

The SC will:

2.1 Coordinate communication among the SS, SP, FVS, and patients at each site, reporting to the CMC.

2.2 Participate in patient recruitment and obtain informed consent...

2.3 Input key information to randomize patients utilizing a simple web-based computer program.

2.4 Assist patients in completion of study questionnaires.

2.5 Collect other relevant data, including historical data, hospital records and reports.

2.6 Assist patients during clinical visits.

2.7 Coordinate the booking for presurgery consultation, confirm the surgery time and the availability of the pathologists and FVS. .

2.8 Prepare and pick up the surgical and pathology packages.

2.9 Process samples: ship the frozen samples, blood samples and recuts of the surgical blocks to the CMC. Request H & E slides and paraffin blocks, or sections from site pathologist for molecular analysis and mail to CMC.

2.10 Coordinate patient follow-up: establish and confirm appointments, collect follow-up tracking sheets, images and path reports, if any. Arrange for CT scans and comparative biopsy at 2-year visit.

2.11 Process collected data: Key in the survey data, scan and upload other relevant data from tests (path report, operation report and MRI or CT), clinical images, surgery margin data from surgery tracking sheet, and pathology tracking sheet and path reports.

2.12 Help CMC to coordinate meetings or teleconferences at each site.

3. FV Specialist (FVS):

The FVS will be trained in using FV. It is suggested this person has a dental background (e.g. a dentist in the cancer centre or an experienced dental hygienist). Another ENT colleague might be suitable. It is imperative this person be readily accessible (clinical time is flexible and flexible for attending surgery). Must be a strong supporter of the proposed research.

The FVS will:

3.1 Perform pre-surgery consultation, including clinical assessment, FV assessment, and imaging.

3.2 Attend the surgery in OR: It estimated that this will require 30 minutes per patient including waiting time, imaging of clinical lesion, FV assessment and imaging, and recording of the surgical margin information.

3.3 Perform follow-ups: Clinical assessment, FV assessment, Imaging (optional: this can be done in SS's office).

3.4 Attend regular teleconference for quality assurance.

4. Site Pathologist (SP):

The study pathologist is an integral part of the team and should have a good understanding of the clinical importance of the project. Ideally, this individual will already have a good working relationship with the SS. He or she will be involved in all aspects of dealing with the tissue after it has been excised.

The SP will:

4.1. Take possession of the specimen immediately following excision, in order to:

- Take images of the specimen using a Polaroid camera
- Collect frozen tissue sample and put into tissue mould with OCT compound and place in dry ice foam box (page SC for pickup)

4.2. On the second day, mark the tissue blocking on the Polaroid image

4.3. Provide an extra set of H and E stained slides to CMC; in order to assess margins (one of the end points).

4.4. Provide CMC the blocks and H & E recuts for review. Blocks of interest (6-10 blocks) will be sectioned for aim#3 analysis as planned and returned to the study sites within a month.

4.5. Participate in regular teleconference for quality assurance.

REFERENCES

1. Parkin DM, Bray F, Ferlay J, et al: Global cancer statistics, 2002. CA Cancer J Clin 55:74-108, 2005

2. Dhooge IJ, De Vos M, Van Cauwenberge PB: Multiple primary malignant tumors in patients with head and neck cancer: results of a prospective study and future perspectives. Laryngoscope 108:250-6, 1998

3. Sardi I, Franchi A, Ferriero G, et al: Prediction of recurrence by microsatellite analysis in head and neck cancer. Genes Chromosomes Cancer 29:201-6, 2000

4. Poh CF, MacAulay CE, Zhang L, et al: Tracing the "at-risk" oral mucosa field with autofluorescence: steps toward clinical impact. Cancer Prev Res (Phila) 2:401-4, 2009

5. Braakhuis BJ, Tabor MP, Kummer JA, et al: A genetic explanation of Slaughter's concept of field cancerization: evidence and clinical implications. Cancer Res 63:1727-30, 2003

6. Slaughter DP, Southwick HW, Smejkal W: Field cancerization in oral stratified squamous epithelium; clinical implications of multicentric origin. Cancer 6:963-8, 1953

7. Brennan JA, Mao L, Hruban RH, et al: Molecular assessment of histopathological staging in squamous-cell carcinoma of the head and neck. N Engl J Med 332:429-35, 1995

8. Leemans CR, Tiwari R, Nauta JJP, et al: Recurrence at the Primary Site in Head and Neck-Cancer and the Significance of Neck Lymph-Node Metastases as a Prognostic Factor. Cancer 73:187-190, 1994

9. Tabor MP, Brakenhoff RH, Ruijter-Schippers HJ, et al: Multiple head and neck tumors frequently originate from a single preneoplastic lesion. American Journal of Pathology 161:1051-1060, 2002

10. Gillenwater A, Jacob R, Ganeshappa R, et al: Noninvasive diagnosis of oral neoplasia based on fluorescence spectroscopy and native tissue autofluorescence. Archives of Otolaryngology-Head & Neck Surgery 124:1251-1258, 1998

11. Heintzelman DL, Utzinger U, Fuchs H, et al: Optimal excitation wavelengths for in vivo detection of oral neoplasia using fluorescence spectroscopy. Photochemistry and Photobiology 72:103-113, 2000

12. Ingrams DR, Dhingra JK, Roy K, et al: Autofluorescence characteristics of oral mucosa. Head and Neck-Journal for the Sciences and Specialties of the Head and Neck 19:27-32, 1997

13. Lam S, Macaulay C, Palcic B: Detection and Localization of Early Lung-Cancer by Imaging Techniques. Chest 103:S12-S14, 1993

14. Muller MG, Valdez TA, Georgakoudi I, et al: Spectroscopic detection and evaluation of morphologic and biochemical changes in early human oral carcinoma. Cancer 97:1681-1692, 2003

15. Ramanujam N, Mitchell MF, Mahadevan A, et al: In-Vivo Diagnosis of Cervical Intraepithelial Neoplasia Using 337-Nm-Excited Laser-Induced Fluorescence. Proceedings of the National Academy of Sciences of the United States of America 91:10193-10197, 1994

16. Zeng HS, McLean DI, MacAulay C, et al: Autofluorescence properties of skin and applications in dermatology. Biomedical Photonics and Optoelectronic Imaging 4224:366-373, 2000

17. Chang SK, Follen M, Malpica A, et al: Optimal excitation wavelengths for discrimination of cervical neoplasia. Ieee Transactions on Biomedical Engineering 49:1102-1111, 2002

18. Collier T, Follen M, Malpica A, et al: Sources of scattering in cervical tissue: determination of the scattering coefficient by confocal microscopy. Applied Optics 44:2072-2081, 2005

19. Drezek R, Guillaud M, Collier T, et al: Light scattering from cervical cells throughout neoplastic progression: influence of nuclear morphology, DNA content, and chromatin texture. Journal of Biomedical Optics 8:7-16, 2003

20. RichardsKortum R, SevickMuraca E: Quantitative optical spectroscopy for tissue diagnosis. Annual Review of Physical Chemistry 47:555-606, 1996

21. Ramanujam N: Fluorescence spectroscopy of neoplastic and non-neoplastic tissues. Neoplasia 2:89-117, 2000

22. Follen M, Crain S, MacAulay C, et al: Optical technologies for cervical neoplasia: update of an NCI program project grant. Clin Adv Hematol Oncol 3:41-53, 2005

23. Andersson-Engels S, Klinteberg C, Svanberg K, et al: In vivo fluorescence imaging for tissue diagnostics. Phys Med Biol 42:815-24, 1997

24. Betz CS, Mehlmann M, Rick K, et al: Autofluorescence imaging and spectroscopy of normal and malignant mucosa in patients with head and neck cancer. Lasers Surg Med 25:323-34, 1999

25. Gillenwater A, Jacob R, Ganeshappa R, et al: Noninvasive diagnosis of oral neoplasia based on fluorescence spectroscopy and native tissue autofluorescence. Arch Otolaryngol Head Neck Surg 124:1251-8, 1998

26. Schantz SP, Kolli V, Savage HE, et al: In vivo native cellular fluorescence and histological characteristics of head and neck cancer. Clin Cancer Res 4:1177-82, 1998

27. Svistun E, Alizadeh-Naderi R, El-Naggar A, et al: Vision enhancement system for detection of oral cavity neoplasia based on autofluorescence. Head Neck 26:205-15, 2004

28. Lane PM, Gilhuly T, Whitehead P, et al: Simple device for the direct visualization of oral-cavity tissue fluorescence. J Biomed Opt 11:024006, 2006

29. Poh CF, Ng SP, Williams PM, et al: Direct fluorescence visualization of clinically occult high-risk oral premalignant disease using a simple hand-held device. Head and Neck-Journal for the Sciences and Specialties of the Head and Neck 29:71-76, 2007

30. Poh CF, Zhang LW, Anderson DW, et al: Fluorescence visualization detection of field alterations in tumor margins of oral cancer patients. Clinical Cancer Research 12:6716-6722, 2006

31. Rosin MP, Cheng X, Poh C, et al: Use of allelic loss to predict malignant risk for low-grade oral epithelial dysplasia. Clinical Cancer Research 6:357-362, 2000

32. Rosin MP, Lam WL, Poh C, et al: 3p14 and 9p21 loss is a simple tool for predicting second oral malignancy at previously treated oral cancer sites. Cancer Research 62:6447-6450, 2002

33. Hayward JR, Regezi JA: Oral dysplasia and in situ carcinoma: clinicopathologic correlations of eight patients. J Oral Surg 35:756-62, 1977

34. World Health Organization. ICD-10 : international statistical classification of diseases and related health problems : tenth revision, 2nd ed. 2004

35. Shaw JW, Johnson JA, Coons SJ: US valuation of the EQ-5D health states - Development and testing of the D1 valuation model. Medical Care 43:203-220, 2005

36. EuroQoL, 2010

37. List MA, D'Antonio LL, Cella DF, et al: The Performance Status Scale for Head and Neck Cancer Patients and the Functional Assessment of Cancer Therapy-Head and Neck Scale. A study of utility and validity. Cancer 77:2294-301, 1996

38. Functional Assessment of Chrnoic Illness Therapy, 2010

39. Rinkel RN, Verdonck-de Leeuw IM, van Reij EJ, et al: Speech Handicap Index in patients with oral and pharyngeal cancer: better understanding of patients' complaints. Head Neck 30:868-74, 2008
