# Supplementary material for: Effect of Fluorescence Visualization–Guided Surgery on Local Recurrence of Oral Squamous Cell Carcinoma: A Randomized Clinical Trial
Source: JAMA Otolaryngol Head Neck Surg. 2020 Oct 8;146(12):1149–55. doi: 10.1001/jamaoto.2020.3147 (PMC7545352; doi:10.1001/jamaoto.2020.3147)
Supplement: Supplement 2. — eTable. Adverse Events, Number of Patients (Events) [file jamaotolaryngolheadnecksurg-e203147-s002.pdf]

## Supplementary Online Content

Durham JS, Brasher P, Anderson DW, et al. Effect of fluorescence visualization–guided surgery on local recurrence of oral squamous cell carcinoma: a randomized clinical trial. *JAMA Otolaryngol Head Neck Surg*. Published online October 8, 2020. doi:10.1001/jamaoto.2020.3262

**eTable.** Adverse Events, Number of Patients (Events)

This supplementary material has been provided by the authors to give readers additional information about their work.

© 2020 Durham JS et al. *JAMA Otolaryngology-Head & Neck Surgery*.

**eTable.** Adverse events, number of patients (events)

| AE | Grade 1 | Grade 2 | Grade 3 | Grade 4 | Grade 5 |
|---|---|---|---|---|---|
| **WL-guided (n=216)** | 192 (792) | 102 (195) | 19 (31) | 1 (1) | 38 (38) |
| **FV-guided (n=227)** | 200 (804) | 125 (219) | 24 (37) | 0 (0) | 41 (41) |

Grade 1: Mild; asymptomatic or mild symptoms; clinical or diagnostic observations only; intervention not indicated. Grade 2: Moderate; minimal, local or noninvasive intervention indicated; limiting age-appropriate instrumental activities of daily living (ADL). Grade 3: Severe or medically significant but not immediately life-threatening; hospitalization or prolongation of hospitalization indicated; disabling; limiting self care ADL. Grade 4: Life-threatening consequences; urgent intervention indicated. Grade 5: death related to adverse events (AE).

Abbreviations: WL, white light; FV, fluorescence visualization

© 2020 Durham JS et al. *JAMA Otolaryngology-Head & Neck Surgery*.
