# Supplementary material for: Effect of Fluorescence Visualization–Guided Surgery on Local Recurrence of Oral Squamous Cell Carcinoma: A Randomized Clinical Trial
Source: JAMA Otolaryngol Head Neck Surg. 2020 Oct 8;146(12):1149–55. doi: 10.1001/jamaoto.2020.3147 (PMC7545352; doi:10.1001/jamaoto.2020.3147)
Supplement: Supplement 3. — Data Sharing Statement [file jamaotolaryngolheadnecksurg-e203147-s003.pdf]

Data Sharing Statement

Durham. Effect of Fluorescence Visualization-Guided Surgery on Local Recurrence of Oral Squamous Cell Carcinoma. *JAMA Otolaryngol Head Neck Surg*. Published October 08, 2020. 10.1001/jamaoto.2020.3147

Data
Data available: No
